# Location, location, location: mapping the lymphoma tumor microenvironment using spatial transcriptomics

**DOI:** 10.3389/fonc.2023.1258245

**Published:** 2023-10-04

**Authors:** Keir Pickard, Emily Stephenson, Alex Mitchell, Laura Jardine, Chris M. Bacon

**Affiliations:** ^1^ Biosciences Institute, Newcastle University, Newcastle upon Tyne, United Kingdom; ^2^ Haematology Department, Freeman Hospital, Newcastle upon Tyne Hospitals NHS Foundation Trust, Newcastle upon Tyne, United Kingdom; ^3^ Translational and Clinical Research Institute, Newcastle University, Newcastle upon Tyne, United Kingdom; ^4^ Department of Cellular Pathology, Newcastle upon Tyne Hospitals NHS Foundation Trust, Newcastle upon Tyne, United Kingdom

**Keywords:** lymphoma, spatial transcriptomics, tumor microenvironment, single cell RNA sequencing, personalized medicine

## Abstract

Lymphomas are a heterogenous group of lymphoid neoplasms with a wide variety of clinical presentations. Response to treatment and prognosis differs both between and within lymphoma subtypes. Improved molecular and genetic profiling has increased our understanding of the factors which drive these clinical dynamics. Immune and non-immune cells within the lymphoma tumor microenvironment (TME) can both play a key role in antitumor immune responses and conversely also support lymphoma growth and survival. A deeper understanding of the lymphoma TME would identify key lymphoma and immune cell interactions which could be disrupted for therapeutic benefit. Single cell RNA sequencing studies have provided a more comprehensive description of the TME, however these studies are limited in that they lack spatial context. Spatial transcriptomics provides a comprehensive analysis of gene expression within tissue and is an attractive technique in lymphoma to both disentangle the complex interactions between lymphoma and TME cells and improve understanding of how lymphoma cells evade the host immune response. This article summarizes current spatial transcriptomic technologies and their use in lymphoma research to date. The resulting data has already enriched our knowledge of the mechanisms and clinical impact of an immunosuppressive TME in lymphoma and the accrual of further studies will provide a fundamental step in the march towards personalized medicine.

## 1 Introduction

Lymphoma is an umbrella term for an incredibly heterogenous group of disorders, with the latest World Health Organization (WHO) classification listing over 100 subtypes. These are broadly categorized into B-cell, T-cell and NK-cell lymphoid proliferations and lymphomas, with further subcategorization based on clinicopathologic, molecular, and genetic data (1). Common to all lymphomas is the important clinical distinction between high-grade and low-grade disease; the natural history and response to treatment vary greatly between these two groups.

Lymphoma tumors reside in a complex ecosystem – the tumor microenvironment (TME) – consisting of malignant cells, immune cells, stromal cells, blood vessels, and the extracellular matrix. The composition of the TME is determined by the interaction between the malignant cell and inflammatory host response, with distinctive TME patterns found in specific subtypes of lymphoma (2). These TME patterns show variable immune cell infiltration and in line with other cancers it is recognized that these immune cells, rather than simply representing an attempt by the immune system to eradicate lymphoma, can in fact enhance and support tumor growth and promote immune evasion with the support of stromal components of the TME (3). There is a growing appreciation of the importance of tissue-resident immune cells in remodeling the TME, including through the induction of tissue-specific tertiary lymphoid structures (TLS) which may support antitumor immune responses; the presence of TLSs in some solid cancers correlates with improved response to immune checkpoint blockade therapy (4). Detailed characterization of tissue-resident immune cells in lymphoma and their influence on prognosis and treatment response is lacking.

Treatment options for lymphoma are varied and selecting the most appropriate strategy requires a holistic assessment of the patient and their lymphoma subtype, including molecular and genetic abnormalities of the tumor. Broad classes of treatment include chemotherapy, radiotherapy, immunotherapy, and targeted molecular therapies. Frontline therapy for lymphoma typically involves chemoimmunotherapy regimens with or without the addition of radiotherapy. In those patients who fail to respond or who relapse following this approach, there is increasing focus on novel cellular-based therapies which redirect the immune system to initiate cell death. Bispecific antibodies are molecules with two different antigen-binding sites which bypass immune evasion and redirect and engage T cells to lyse malignant cells (5). In lymphoma the current bispecific antibodies in clinical use have binding sites for CD3+ T cells and CD20+ tumor antigens and have demonstrated efficacy in B cell lymphoma (6, 7). Chimeric antigen receptor (CAR) T cell therapy, whereby autologous T cells are isolated and engineered to specifically target antigens on the malignant cell surface (e.g. CD19 on B cell lymphoma) is established in the treatment of B cell lymphomas including diffuse large B cell lymphoma (DLBCL), follicular lymphoma and mantle cell lymphoma (8–13). Targeted molecular therapies are also used in the second or subsequent line of therapy. They function by exploiting specific vulnerabilities in cancer cells, for example Bruton’s tyrosine kinase inhibitors (BTKi) in mantle cell lymphoma (14).

Immunotherapies have also been developed which target the lymphoma TME. Perhaps the best established example is a group of drugs termed checkpoint inhibitors which target and block checkpoint molecules - negative regulators of T cell activation which include programmed cell death 1 (PD1) (15). Tumor cells can upregulate PD1 ligands to induce T cell exhaustion and mold the TME towards a supportive niche; blocking the PD1 axis with PD1 inhibitors (e.g. pembrolizumab and nivolumab) has been shown to have substantial anti-tumour activity in relapsed or refractory Hodgkin lymphoma (16, 17), primary mediastinal B cell lymphoma (18) and also in T cell (19, 20) and NK/T cell lymphoma (21). However the initial effectiveness and duration of response of checkpoint blockade differs both between and within lymphoma subtypes.

A deeper understanding of the lymphoma TME is required to identify key interactions which could be disrupted for therapeutic benefit and to dissect the key players in lymphoma immune evasion, thereby improving our understanding of the variability in response rates and duration of response to immunotherapy seen in different subtypes of lymphoma. This information would contribute to the treatment paradigm shifting away from a ‘one size fits all’ chemotherapy approach and towards precision medicine. Understanding of the spatial context of the lymphoma TME primarily evolved through techniques such as histology, immunohistochemistry and immunofluorescence microscopy. Advances are continuously being made in multiplexed imaging techniques, such as the Co-Detection by indEXing (CODEX) platform which can visualize up to sixty DNA-conjugated antibodies (22). Characterization of the tumor and immune cell subpopulations within the TME can be studied using proteomic techniques such as mass cytometry or cytometry by time-of-flight (CyTOF) (23). These techniques offer valuable information on the spatial distribution of protein expression, but remain limited in the number of parameters studied within a single experiment (24) and are not spatial transcriptomic technologies and so will not be explored further in this article.

The ideal method to explore the TME would be a high-dimensional technology, combined with spatial information as to where particular cells are located and to which cells they are in close proximity. In this article we will explore one such technique, spatial transcriptomics, and review existing studies which harness this technology to better understand the lymphoma TME and the translational impact of the data generated.

## 2 Introduction to spatial transcriptomic technologies

Spatial transcriptomic technologies were developed to address the loss of spatial context when performing high resolution transcriptome analyses. For example, single cell transcriptomics usually requires the dissociation of tissues into a single cell or single nuclear suspension, which will disrupt the organization of cells within tissues. Single cell RNA sequencing (scRNAseq) allows for the unbiased analysis of cellular identity so can resolve heterogeneity within cell types (25). However, without knowing the position of cells in relation to other cells or structures, other techniques must be used to explore the cellular architecture of tissues. Spatial transcriptomics can measure gene activity whilst mapping where in the tissue this activity is occurring.

**Figure 1:**
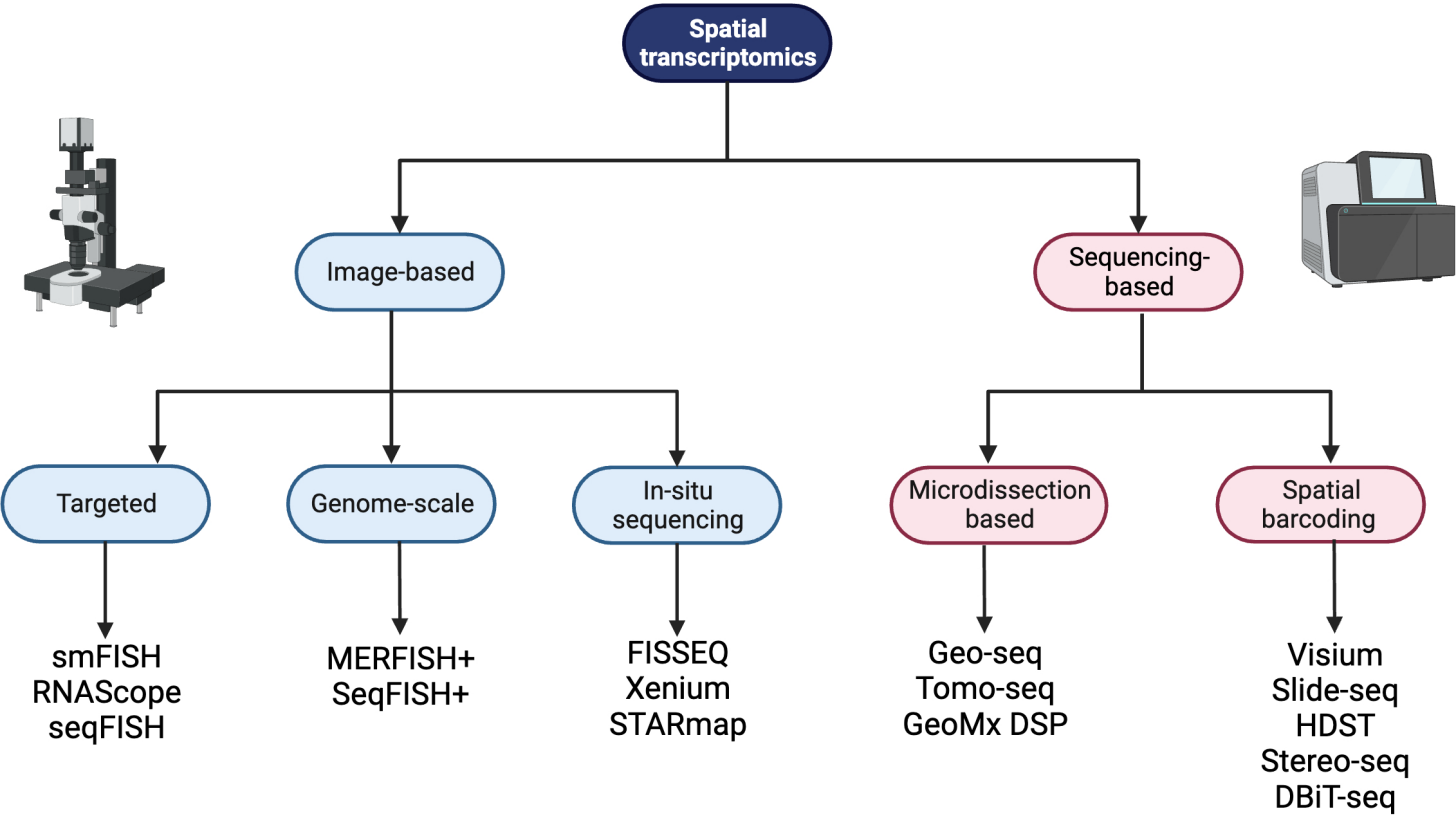
A summary of spatial transcriptomic technologies divided into image-based and sequencing-based technologies, with subdivisions based on the techniques employed. Created with BioRender.com.

Spatially resolved transcriptomics was named Nature Method of the Year for 2020 and the approaches to generate and analyze the data are rapidly developing (26). Spatial transcriptomic techniques can be broadly separated into two omics-based categories; image-based or next generation sequencing (NGS) based approaches, and some of the available technologies are summarized in **Figure 1**. The selection of methods for spatial transcriptomics will depend on the experimental aim and the balance between the number of detected transcripts and spatial resolution, as well as other factors such as cost, size and preservation method of tissue, and access to image-processing and bioinformatic pipelines. **Table 1** compares key features between the established techniques of spatial transcriptomics.

**Table 1:** A comparison of important features of key spatial transcriptomic techniques currently in wide use.

Technology	Technique	Aim of study	Efficiency of transcript detection	Transcriptome-wide or targeted profiling	Single cell	Tissue area
Image-based	smFISH e.g. RNAScope	Hypothesis testing	High	Targeted	Yes	Limited
	ISS e.g. Xenium 10x Genomics	Hypothesis testing	Low	Targeted	Yes	Limited
Sequencing- based	Barcoding e.g. Visium 10x Genomics	Hypothesis generating	Low	Transcriptome-wide	No	Large
	ROI e.g. GeoMx DSP NanoString	Hypothesis generating	Low	Transcriptome-wide	Yes	Limited by ROI

smFISH, single molecule fluorescence in situ hybridisation; ISS, in situ sequencing; ROI, region of interest; DSP, digital spatial profiler.

### 2.1 Image-based spatial transcriptomic technologies

Image-based spatial transcriptomic technologies use microscopes to detect and quantify the RNA within the tissue. Nucleic acids are labeled with complementary fluorescent probes using *in situ* hybridization (ISH) which is an established technology within the clinical setting (27); for example c-MYC protein expression is routinely used in the clinical diagnosis and prognosis of aggressive B-cell lymphomas (28). A great advantage of ISH is it can generate spatial data about gene expression and genetic loci (29). Immunohistochemistry (IHC) can also complement ISH data by increasing the accuracy of cell type identification.

When ISH first became available it was largely qualitative, until single molecule fluorescence ISH (smFISH) emerged allowing each transcript to be labeled and quantified. The technique usually requires multiple DNA probes that have the same fluorescent tag and are all complementary to the target transcript. This increases the signal and reduces any signal-to-noise ratio (30).

There are many forms of ISH that are available today. They can either be targeted to specific RNA transcripts or provide genome scale measurements. RNAScope is a commercially available ISH assay that works on both fresh frozen (FF) and formalin-fixed paraffin-embedded (FFPE) tissue sections. The technology has vastly improved the sensitivity and specificity compared to standard smFISH whilst increasing signal detection. It works by using “double-Z” shaped probes which bind specifically to the RNA molecules and allow for sequential hybridizations to amplify the signal. Multiplexing is possible for up to twelve targets from FFPE tissue and up to forty-eight targets from FF tissue. The readout is detected on an epifluorescent microscope (31). The drawback of RNAScope is the limited number of genes that can be targeted in one experiment. Two technologies have sought to address this: seqFISH+ and MERFISH. Both methods can target thousands or tens of thousands of genes in one experiment, by using combinatorial labeling and sequential imaging with each having a slightly different probe design. However both come with a high price tag and, at the moment, they are both still only available for FF tissue (32, 33).

*In situ* sequencing (ISS) is another image-based spatial technology that employs a similar concept of ISH but uses a unique padlock probe design and rolling circle amplification to amplify the DNA of bound probes. This amplified cDNA is then sequenced *in situ* by further ligation of probes which are fluorescently labeled to enable signal readout (34). The ISS technology has been commercialized on a number of occasions with the original protocol under the name of FISSEQ (35). It is now available through 10x Genomics’ Xenium platform which has automated much of the process. Although another costly approach, the commercialization has the added benefit of customer support and dedicated analysis tools. STARmap has expanded on the ISS approach in 3D intact tissue but these experiments can require long capture times which are not yet fully automated (36).

### 2.2 Sequencing-based spatial transcriptomic technologies

Sequencing-based spatial transcriptomic technologies use DNA sequencers to detect the spatially resolved transcripts in the tissue. There are two main methods by which this can be achieved. Firstly, laser-capture microdissection (LCM) has been adapted to identify and isolate regions of interest on tissue sections and analyze their RNA content. Geo-seq enables this to be done at single cell level (37) whilst Tomo-seq profiles genome-wide expression of 50-100 μm cryosections (38). The most contemporary method is the commercialized GeoMx DSP platform from Nanostring. This can also allow for single cell level profiling and works with both FF and FFPE tissue sections. Specific transcripts are bound with probes that are cleaved and sequenced at regions of interest. The number of sequencing libraries is dependent on the number of regions of interest and so cost is variable (39).

The other approach is to capture mRNA from the tissue directly by using space-specific barcoded oligos, in an array-like fashion, so that their location is retained. Spatial Transcriptomics (ST) was the first method to employ this design using glass slides with barcodes directly situated on top. FF tissue sections are mounted directly on the slides and the tissue is permeabilized to release the mRNA, which is then subsequently barcoded and captured via the polyA tail. cDNA is created on the surface of the slide and then cleaved, so that NGS-ready libraries can be prepared (40). ST is another method that has been commercialized by 10x Genomics (Visium), who have increased resolution by decreasing the barcoded areas from 100 μm to 55 μm, whilst also making the method available for FFPE tissue via probe capture rather than polyA. To complement the Visium FFPE method, a slide transfer instrument, called CytAssist, enables researchers to automate the transfer of material from pre-mounted sections to the proprietary Visium slides.

The limitation to the Visium platform is still resolution and other technologies have sought to improve this. Slide-seqV2 and HDST have a resolution of 10 μm and 2 μm respectively and both use barcoded beads at each capture location on the slide to increase gene recovery (41, 42). Stereo-seq has achieved an even greater resolution of 0.5 μm by the addition of wells onto chips patterned with DNA-nanoballs to capture RNA *in situ* which is then reverse transcribed on the chip and cleaved for further library preparation (43). Stereo-seq is currently in the early stages of commercialization by BDI and the company has pledged to keep prices low.

Deterministic barcoding in tissue for spatial omics sequencing (DBiT-seq) is currently the only technology available that delivers barcodes to the tissue itself using microfluidics. The capture area can be flexible and can range from 10, 25, or 50 μm, which can be useful when profiling tissues with homogeneous regions (44). At the moment, the great advantage of DBiT-seq is that it is easily combined with protein detection by using oligo conjugated antibodies, however some say the method is labor intensive and throughput is fairly low.

**Figure 2:**
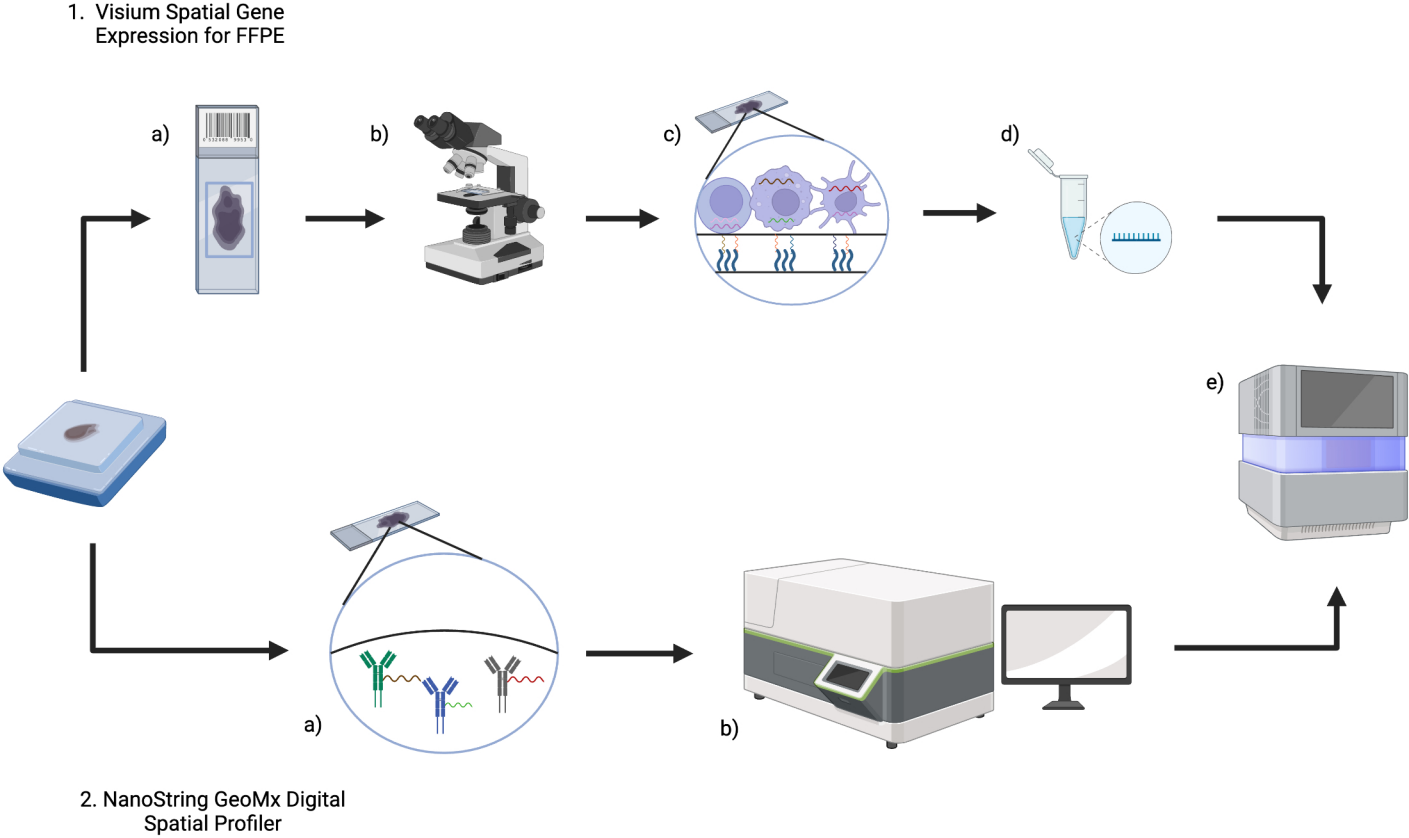
An overview of Visium (10x Genomics) and GeoMx Digital Spatial Profiler (DSP) (NanoString) technologies for FFPE tissue. 1. For Visium, (a) sections from an FFPE tissue block are placed on a Visium Gene Expression slide and then b imaged (either H&E or IF) to provide histological context and for downstream analysis. c The Visium slide contains capture areas where capture probes will bind to RNA and incorporate spatial barcodes, before d preparation of libraries for e next generation sequencing. 2. In GeoMx DSP, a FFPE blocks are sectioned onto slides and then DSP barcoded probes will bind RNA (and/or protein). b The tissue section can then be imaged and reviewed to select a region of interest, before e) measuring count expression levels within the region of interest using next generation sequencing. Created with BioRender.com.

As will be outlined in subsequent sections, the Visium (10x Genomics) and GeoMx DSP (Nanostring) commercial platforms are established in lymphoma research and **Figure 2** summarizes the workflow of these technologies.

### 2.3 Analysis of spatial transcriptomic data

The aim of spatial transcriptomic data analysis is to assimilate gene expression data with spatial locations to obtain useful biological insights. Spatial transcriptomic data analysis will vary depending on the technology employed and the final data output (e.g. microscope images or sequence reads), and each experiment requires a bespoke approach. The bioinformatic analysis of spatial transcriptomic data is an expert and rapidly moving field; although a detailed review of the complexities and approaches for each technology is beyond the scope of this article, this section will introduce some key concepts in data analysis. Common steps include image preprocessing, normalization of data, integration of scRNAseq data for cell-type deconvolution and further downstream analysis including inference of cell-to-cell interactions (45).

Initial preprocessing steps are similar to the quality control considerations in a typical scRNAseq experiment and include normalization to account for the variance in sequencing depth across spots. However cell density can vary considerably across a tissue and it is important to account and normalize for technical artifacts without losing data which represents true biological variance. For image-based spatial transcriptomics (e.g. smFISH- and ISS-based methods), key aspects of preprocessing include image registration, high-throughput transcript signal detection and localization, and then cell segmentation. For sequencing-based spatial transcriptomics (e.g. Visium), steps include processing and dividing the original tissue image, aligning transcripts to reference genomes and then combining these outputs. The resulting data output for both image- and sequencing-based technologies is a gene-by-cell count matrix alongside a location matrix of cell coordinates (46).

The data from spatial transcriptomics is very complex, and dimensionality reduction techniques such as principal component analysis (PCA) or manifold learning are required to visualize the data within a 2D space and to cluster cells with similar transcriptomes, utilizing methods such as agglomerative clustering (47). These workflows are similar to those utilized in scRNAseq experiments, but with the clear advantage that the results can be overlaid onto the tissue image to allow visualization of gene expression data in the histological context. Spatially variable features can then be identified based on differential expression in anatomical regions within the tissue (48).

**Figure 3:**
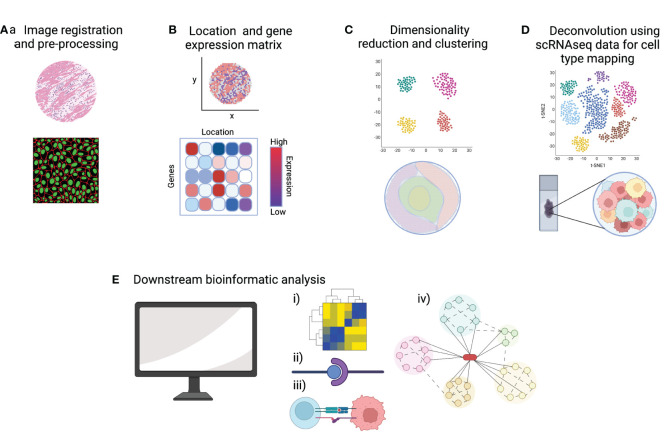
A summary of key steps of data analysis in spatial transcriptomics. **(A)** Pre-processing steps include quality control (QC) metrics and image registration. **(B)** A common starting point for downstream bioinformatic analysis is a gene-by-cell count matrix and a matrix of cell coordinates. **(C)** Normalization, dimensionality reduction and clustering allows the visualization of gene expression data in 2D and overlaid onto the tissue image. **(D)** Integration of scRNAseq data allows deconvolution and cell type mapping of the spatial transcriptomic data to allow visualization of cell locations within the spatial context of the original tissue section. **(E)** Bioinformatic pipelines can then be used to explore key aspects of cell signaling and cell-microenvironment analysis such as (i) cell-to-gene interactions, (ii) ligand-receptor pairing, (iii) cell-to-cell interactions and (iv) trajectory analysis. Created with BioRender.com.

As discussed in the previous section, many whole-transcriptome spatial transcriptomic technologies do not yet offer single-cell resolution, which means each spot will likely include multiple cells. Existing scRNAseq datasets, matched to the particular tissue/pathology, are often integrated with the spatial transcriptomic data to predict which cells are present within each spot. Methods to integrate data include mapping and deconvolution. Mapping is used more frequently in image-based spatial transcriptomic methods to assign annotations established in scRNAseq data to spatial locations in the tissue section. Deconvolution strategies are more commonly employed in sequencing-based technologies by calculating the probabilities that specific cell-type transcriptomes are represented within each capture area (46). The user can then characterize and visualize spatial patterns of tissue cellular heterogeneity including the proximity of cells to each other. Further downstream analysis includes exploring potential cell-to-cell interactions, inferred ligand-receptor pairings and cell trajectory analysis. The resulting spatial network patterns and defined cellular neighborhoods are of great interest when considering lymphoma cells within their tumor microenvironment (49). **Figure 3** summarizes the bioinformatic workflow in spatial transcriptomic analysis.

A current difficulty in approaching and critically appraising the bioinformatic analysis of spatial transcriptomic data is the huge variety in computational approaches which exist and which continue to grow; for scRNAseq analysis alone there are over 1500 software analysis tools (50). The fact that distinct research groups will analyze and hold data in different formats limits the reproducibility of results and reusability of the data and an important future direction will be the streamlining and perhaps even standardization of bioinformatic pipelines to ensure a consistent and clinically-relevant approach. As a bare minimum, researchers should be encouraged to provide open access regarding methods and interoperability of data; the growing field of research software engineering will play a crucial role in supporting this process (51). A further issue is the size of the data generated, particularly if spatial transcriptomic data is combined with other modalities such as proteomics and chromatin accessibility; storage methods and processing speeds can be problematic even in large, established laboratories (52).

## 3 Spatial transcriptomic analysis in lymphoma: studies to date

The following section will review the use of spatial transcriptomic techniques in distinct lymphoma subtypes, followed by a discussion of the limitations of the technology, and future directions of lymphoma research. **Table 2** summarizes the current, published studies which have utilized spatial transcriptomic technologies to improve our understanding of the lymphoma tumor microenvironment.

**Table 2:** A summary of currently published studies utilizing spatial transcriptomics in lymphoma, including key discoveries from the dataset.

Lymphoma subtype	Technology	Reference	Tissue	No. of samples	Region of interest selection	Discovery
Hodgkin lymphoma	Nanostring GeoMx DSP	Stewart et al., 2023 (53)	FFPE	10	CD274/PDCD1 (PD-L1)	Enrichment of distinct and recurring tissue niches within cHL lymph nodes, including MNP networks, are associated with patient outcome
DLBCL	NanoString GeoMx DSP	Sangaletti et al., 2020 (54)	FFPE	8	CD20/CD271/SMA	Intra-tumour heterogeneity exists in immune and stromal networks in DLBCL which correspond to MYC expression
DLBCL	NanoString GeoMx DSP	Liu et al., 2023 (55)	TMA (tissue microarray)	47	CD68/CD3/CD20	Macrophage subsets in DLBCL are spatially located. Certain macrophage signatures are prognostic and could be therapeutically targeted
PCNSL	10x Genomics Visium	Heming et al., 2022 (56)	FFPE	4	N/A	Spatial distribution of malignant B cell niches enhances local immunosuppression and contributes to therapy resistance
PCNSL	10x Genomics Visium	Xia et al., 2023 (57)	FFPE	4	TME hot/cold	PCNSL tumor cells evolve through a TME remodeling pattern, with FKBP5+ cells contributing to a barrier effect; tumor subgroups have their own spatial functional zones
Follicular lymphoma	10x Genomics Visium	Attaf et al., 2022 (58)	Fresh frozen	1	N/A	Distinct malignant B cell states localized to specific niches within the lymph node follicle
AITL	10x Genomics Visium	Du et al., 2022 (59)	Fresh frozen	1	N/A	The TME of AITL is immune-suppressive, with upregulation of CCR4 and its ligands CCL17 and CCL22.

DLBCL, diffuse large B cell lymphoma; PCNSL, primary central nervous system lymphoma; AITL, angioimmunoblastic T-cell lymphoma.

### 3.1 Hodgkin lymphoma

Classic Hodgkin lymphoma (cHL) accounts for around 15% of lymphoma diagnoses and presents with a bimodal age distribution, affecting young adults with an additional smaller peak in older adults. Treatment is based on chemotherapy alone or in combination with radiotherapy depending on individual disease burden. The stage of disease at diagnosis will affect the likelihood of cure but typically reported figures range between 60 to 90% (60). As described in the previous section, the addition of PD1 inhibitors improves outcomes in the relapsed or refractory setting, demonstrating the importance of targeting the interaction between tumor cells and the immune system.

cHL has a unique microenvironment due its distinctive cellular organization. A lymph node infiltrated by cHL is predominantly composed of non-malignant immune cells, including numerous T-cells, NK cells, mononuclear phagocytes, monocytes and dendritic cells. The Hodgkin tumor cells are rare and represent only a very small fraction of the lymph node cellularity (2). These tumor cells are able to evade the antitumor immune response and create a tumor-tolerant microenvironment. T and NK cells represent a significant proportion of cells in the microenvironment established by malignant Hodgkin cells, however they are rendered functionally ineffective. Immune system diversion is achieved via the acquisition of regulatory properties, such as LAG3-, PD-1-, and CTLA4-expression on T cells leading to exhausted T cell phenotypes. Moreover, there appears to be a unique spatial arrangement of T-cells such that the most immunosuppressive subpopulations lie in closest proximity to the malignant Hodgkin cells [reviewed in (60)]. Aoki et al. detailed the functional and spatial characteristics of T-cells in cHL at single cell resolution using scRNAseq data complemented with spatial assessments (immunohistochemistry and imaging mass cytometry). They identified a regulatory T-cell like immunosuppressive subset of LAG3+ T cells lying in close proximity to the tumor cells and thus contributing to the immune escape phenotype (61).

Stewart et al. (53) used the Nanostring GeoMx platform to analyze ten FFPE Hodgkin lymphoma lymph nodes. The authors used PD-L1 to identify Hodgkin/Reed-Sternberg cells (HRSCs) and identified regions of interest (ROIs) in both PD-L1^high^ and PD-LI^low^ areas of tissue. Single cell transcriptome profiles of both normal and pathological lymph nodes were used to provide an overview of the cellular ecosystem of HL. These data were then used as a reference to deconvolute the spatial transcriptomic profiles of cells within the distinct ROIs. Two neoplastic PD-L1^high^ clusters were identified which showed divergent localization of immune cells; one cluster of HRSCs was enriched for T helper cells (Th), exhausted CD4+ T-cells (ThExh) and NK-cells, whereas the other was enriched for mononuclear phagocytes (MNPs) including classical monocytes, macrophages and conventional dendritic cells (cDC2). Conventional dendritic cells and monocytes expressed immunoregulatory checkpoint molecules PD-L1, TIM-3 and the tryptophan-catabolizing enzyme IDO1, therefore contributing to the tumor-tolerant microenvironment. Classical monocytes appeared to play an important role in retaining immunosuppressive and phenotypically exhausted T-cells as well as the exclusion of plasmacytoid dendritic cells. Ligand-receptor interactions were interrogated and confirmed the expression of inhibitory molecules by MNPs in close proximity to HRSCs. Correlation of the transcriptional profile with gene expression data showed that high expression of genes associated with the MNP-rich module was correlated to early treatment failure. This study demonstrates the spatial polarization of tumor-associated MNPs to provide an immunoregulatory niche in close proximity to HRSCs and suggests that the inflammatory cDC2-monocyte-macrophage niche is associated with inferior response to treatment. The identification of these spatial tissue niches could allow improved characterisation of HL tumors prior to therapy, and subsequent targeting of therapies to deplete the inflammatory and immunosuppressive MNPs, alongside existing strategies such as PD-1 blockade.

### 3.2 High-grade B cell non-Hodgkin lymphoma

#### 3.2.1 Diffuse large B cell lymphoma

DLBCL is the commonest category of high-grade NHL but is a heterogeneous disease with differing clinical outcomes based on distinct malignant B cell states. Several classifications exist based on cell of origin, gene expression profiling, and bulk genetic and transcriptomic analysis (62–65). ScRNAseq profiling demonstrates a similarly heterogeneous tumor microenvironment (66) with distinct sub-categorizations of the TME identifiable through functional gene expression signatures (67). The composition of the TME has prognostic value (68, 69).

Studies utilizing spatial transcriptomic technologies are limited in DLBCL. Sangaletti et al. used a murine DLBCL model to demonstrate the impact of the stromal microenvironment on lymphoma gene expression and tumor heterogeneity (54). Different types of mesenchymal cell meshworks exist within the same DLBCL tumor, including those rich in the myofibroblastic/reticular cell maker smooth muscle actin (SMA) and the mesenchymal stromal cell/pericytic marker nerve growth factor receptor (NGFR). The immune and stromal composition of spatially-resolved DLBCL microenvironments was investigated using eight human FFPE samples with the NanoString GeoMx Digital Spatial Profiler (DSP) and four distinct ROIs were profiled within each sample based on expression of SMA+ or NGFR+ stromal networks. Differential gene expression analysis suggested that SMA-rich stromal networks were positively enriched in immunoregulatory and vascular stroma-associated transcripts as compared to NGFR-rich stromal networks. The use of spatial data allowed the authors to identify the intra-lesional heterogeneity of mesenchymal foci within the same DLBCL lesion which corresponded to MYC expression; the presence of an NGFR-rich foci correlated to downregulated MYC expression within the same tumor area. It can therefore be hypothesized that the extent and differential selection of these intra-lesional stromal loci could impact disease progression and response to treatment.

Liu et al. used the NanoString GeoMx DSP to comprehensively characterize DLBCL tumor-associated macrophages (TAMs) (55). They identified eight distinct subsets of macrophages with distinct biological characteristics and which localized to different tissue regions. Macrophage signatures in DLBCL included upregulation of CD163, complement system genes and signaling pathways triggered by TNF-alpha via NF-kB, which confer a pro-tumor immunoregulatory transcriptional profile. The spatial localization of distinct macrophage signatures within DLBCL provides a springboard for further work to evaluate their interaction with lymphoma cells and other immune cells within the TME.

#### 3.2.2 Primary central nervous system lymphoma

PCNSL is a rare subtype of non-Hodgkin lymphoma which pathologically resembles DLBCL, but is confined to the CNS. Genetic and molecular studies have shown recurrent driver mutations in PCNSL, many of which are involved in NF-kB signaling (62, 63, 70). Multi-omic data integration reveals molecular subtypes of PCNSL which can be identified through bulk RNA sequencing and which both correspond to distinct tumor microenvironment signatures and correlate with overall survival (67, 71). In the most recent iteration of the WHO classification system, PCNSL is grouped alongside primary large B-cell lymphoma of the vitreoretina and primary large B-cell lymphoma of the testis under the umbrella term ‘large B-cell lymphomas of immune-privileged sites’ (1). The TME and methods of tumor immune evasion in lymphomas arising in immune sanctuaries are distinct from systemic DLBCL, an example being the loss of HLA class I and II expression on PCNSL cells (72). To further elucidate the immune contexture of PCNSL, Alame et al. (73) used bulk RNA sequencing analysis to highlight three distinct immune cell signatures with high, intermediate or low immune gene expression levels; PCNSL tumors lacking an immune-rich TME correlated with inferior clinical outcomes. This study also demonstrated clinically relevant immune checkpoint ligand-receptor interactions, with high PD-L1-expressing tumor associated macrophages (TAMs) and high TIM-3 expression associated with an immune-rich TME and improved outcomes.

Wei et al. (74) performed scRNAseq on fresh PCNSL tissue and demonstrated an immune rich milieu including plentiful infiltrating B cells, T cells, macrophages and dendritic cell populations; single cell analysis allowed greater resolution to determine the sub-clusters of each immune cell type and to infer functional heterogeneity, for example highlighting the potential importance of CD74 in regulating communication between PCNSL cells and T cells, macrophages and dendritic cells.

Heming et al. (56) devised an approach to perform scRNAseq and single-cell B cell receptor signaling (scBCR) on cells released from PCNSL biopsy into surrounding fluid (termed the “Whiskey Method”) from two patients with PCNSL. They identified significant intratumor heterogeneity in both malignant and non-malignant B cells, including differential expression of chemokines and varied patterns of B cell development. T cells featured an increase in those expressing canonical markers of T-cell exhaustion, including immune checkpoint proteins such as TIM-3 and PD1 as described previously, and T cells with a regulatory CD4+ phenotype. Receptor-ligand interactions were predicted between malignant B cells and T cells/myeloid cells and suggested several means through which the tumor could evade the host immune response. Four malignant B cell clusters (mBc) were identified. The mBc4 cluster was characterized by expression of genes involved in cancer proliferation and enhanced expression of immune checkpoint ligands. Heming et al. went on to perform spatial transcriptomics on four patient samples using Visium Spatial Gene Expression for FFPE (10X Genomics) and integrated their scRNAseq data. The malignant B cell clusters demonstrated focal spatial enrichment in all biopsies, and areas dominated by the mBc4 cluster showed increased expression of exhaustion markers (LAG3, PDCD1, HAVCR2, TIGIT) suggesting that this subtype of malignant cell is able to induce a stronger immunosuppressive TME, and supporting the projected ligand-receptor interactions from the scRNAseq data. The demonstration of transcriptional heterogeneity by both dissociated cell and spatially-resolved techniques increases the strength of the hypothesis that tumor cell niches confer local immunosuppression and therapy resistance and will be key in the development of individualized therapy.

Xia et al. (57) used Visium to analyze different categories of TME in PCNSL. They used pathological assessment to identify four PCNSL samples which resembled each of their four classifications; ‘hot’, ‘invasive margin excluded (IME)’, ‘invasive margin immunosuppressed (IMS)’ and ‘cold’. In brief, the hot TME demonstrated wide distribution of T cells, whereas the cold TME featured very few T cells. In IME, few T cells were found inside the tumor, but there was extensive accumulation around the invasive margin, whilst in IMS there were also few T cells in the tumor but less accumulation in the invasive margin compared to IME. Differentially expressed gene (DEG) analysis and gene set variation analysis (GSVA) demonstrated that tumor cell clusters varied in their function in the distinct TMEs. Tumor cells in the ‘hot’ TME were more likely to be in a state of passive immune defense compared to cancer cells in a ‘cold’ TME which were in a state of negative immune regulation. Developmental trajectory analysis of the tumor cell groups allowed the authors to demonstrate a TME remodeling pattern whereby the ‘hot’ tumor pattern appeared to be a starting point leading to either ‘cold’ or ‘IME’ tumors with immune cell depression and dominance of tumor cells, with the IMS TME as a transitional state where the tumor and immune cells are vying for control. This developmental trajectory is influenced by T cell abundance; if there are few cytotoxic T cells in the TME the tumor cells will branch towards a ‘cold’ cell state, whereas if there are abundant T cells the likely route is to the ‘IME’ terminal state with its associated tight junction to block immune cell activity. By identifying the key genes involved in this transition, it may be possible to better understand and potentially reverse this pathway. Converting a tumor back to a ‘hot’ rather than ‘cold’ or ‘IME’ TME could render it more susceptible to immunotherapy and, indeed, unique changes in expression of PD1 and PDL1 showed temporal and spatial heterogeneity within the distinct TMEs. Efficacy of immunotherapy could also be enhanced by targeting the tumor cells which form a barrier environment, and this study postulates FKBP5 as a key gene associated with both this process and tumor progression.

### 3.3 Low-grade B cell non-Hodgkin lymphoma

#### 3.3.1 Follicular lymphoma

Follicular lymphoma is classified as an indolent, low grade lymphoma however the clinical course and response to therapies can be heterogeneous. Poorer outcomes are seen in those patients with progression of disease within 24 months (POD24) (75). Specific features of the TME, including gene expression signatures of immune response, are associated with disease progression and survival in follicular lymphoma (76–79). Mondello et al. (80) utilized IHC, CyTOF and CODEX to demonstrate that a lack of intrafollicular CD4+ expression correlates with poorer outcomes and can be incorporated into risk stratification models. These intrafollicular CD4+ T cells are of the active, non-exhausted central memory phenotype which provide immune response against the lymphoma cells, and the lack of these cells in more aggressive follicular lymphoma may be modulated by aberrant expression of BCL-6 target genes by the lymphoma cells.

Han et al. (81) used their scRNAseq-derived signatures of T-cell subpopulations in follicular lymphoma to reveal four distinct subtypes of TME. The T-cell depleted TME was associated with inferior outcomes and correlates with increased levels of tumour cell MHCII expression and which will likely result in poorer responses to therapy such as immune-checkpoint blockade. Radtke et al. (82) utilized scRNAseq alongside multiplexed antibody-based imaging to further reveal the rich diversity of immune cells within the TME of follicular lymphoma, including rarer populations which can be difficult to identify in bulk RNA sequencing such as endothelial and fibroblast cell subsets. The Iterative Bleaching Extends multipleXity (IBEX) imaging method performed in this study revealed distinct features of the TME in patients with more clinically aggressive disease, including expansion of desmin-positive fibroblasts around the neoplastic B cell follicles and increased proportions of DC-SIGN-positive cells within the follicles, which were in direct communication with IRF4-positive malignant B cells. The reprogramming of lymphoid stromal cells to follicular lymphoma cancer-associated fibroblasts (FL-CAFs) results from bidirectional interaction between malignant FL B cells and lymphoid stromal cells, including upregulation of CXCL12, CCL19 and CCL21, and TGF-β signaling (83).

Attaf et al. (58) used scRNAseq to demonstrate intra-tumour heterogeneity in FL, finding recurrent malignant B cell states which appear to result from functional plasticity in response to TME cues, particularly signaling from Tfh cells. They went on to perform spatial transcriptomic analysis on a single fresh frozen FL lymph node section using Visium Spatial Gene Expression. They focussed their analysis on distinct tissue areas in relation to the malignant tumor follicles and used reference-based deconvolution from their scRNAseq dataset. Distinct malignant B cell states mapped to different tumor areas, with germinal center and memory B cell-like states preferentially localizing to centrofollicular and interfollicular zones respectively. Perifollicular (PF) zones contained multiple malignant B cell states and were abundant in Tfh-activated cell states and markers of follicular dendritic cells (FDCs). These localizations suggest distinct immune and stromal cells promote survival of particular malignant B cell states within distinct tissue niches. This heterogeneity and plasticity in malignant B cell states and their localization with the TME could explain the variable responses to therapy in FL and eventual relapse and represents a future therapeutic avenue.

### 3.4 T cell non-Hodgkin lymphoma

T-cell lymphoma represents a large spectrum of disease subtypes which typically carry inferior prognosis compared to B-cell lymphoma. The World Health Organisation (WHO) classifies T-cell lymphomas into subtypes with peripheral T-cell lymphoma not otherwise specified (PTCL NOS) and angioimmunoblastic T-cell lymphoma (AITL) being the most common (84).

Du et al. used Visium Spatial Gene Expression to evaluate the tumor microenvironment in a single excised fresh frozen lymph node from a patient with AITL (59). AITL is derived from T follicular helper cells (TFH) and the authors defined the original site of disease by CD4-positive TFH cells within the germinal center region (TFH-GC). Existing scRNAseq datasets from lymphoid tissue was used to conduct differential expressed gene analysis and genes encoding CCL17 and CCL22 were significantly upregulated in the TFH-GC region, with spatial colocalization of T-regulatory cells (Treg). There was also an increased proportion of cycling B cells and vascular smooth muscle cells within the core tumor area, with decreased levels of NK cells and CD8+ cytotoxic T cells, suggesting an immunosuppressed TME. Both CCL17 and CCL22 are ligands for CCR4, and recruit Treg cells to the TME as evidenced in cutaneous T cell lymphoma (CTCL) (85). The anti-CCR4 molecule mogamulizumab has shown promise in CTCL (86), and Du et al’s study suggests this mechanism is a potential therapeutic avenue in AITL.

There is no published use of spatial transcriptomics in cutaneous T-cell lymphoma (CTCL). However, Phillips et al. used scRNA-seq and imaging techniques to demonstrate that topographical differences between PD-1-positive T-cells correlates strongly with pembrolizumab response (87). This work highlights the importance of understanding the spatial arrangements of cells within the tumor microenvironment, which could be further evaluated using spatial transcriptomics.

## 4 Limitations of spatial transcriptomics in lymphoma

The holy grail of spatial transcriptomic technologies would be a technique which offers transcriptome-wide profiling at single-cell resolution and with robust and efficient gene detection. However, at present spatial transcriptomic technologies fall short in one or more of these areas and investigators are required to select the technique to best match their tissue and experimental hypothesis (88). The seven studies summarized in **Table 1** include a combined total of seventy five patient samples; clearly the small sample size limits wider translational impact, particularly in the setting of significant heterogeneity both between and within lymphoma subtypes. It could be argued that spatial transcriptomics remains a relatively new technology and so there will likely be a significant increase in studies which utilize this technology in the coming years. However these techniques remain technically complex, both in regards to sample preparation and downstream bioinformatic analysis. Spatial transcriptomic techniques also remain reliant on the selection of histological sections which may under-represent the full complexity and heterogeneity of the tumor or organ in which it resides (89). Expense is an issue, with the high-throughput processes and comprehensive gene capture of the resulting data reflected in higher price brackets compared to traditional methods such as IHC and IF. It could be argued that spatial transcriptomics remains a hypothesis-generating area of discovery research. The studies in **Table 1** utilized protein level validation methods alongside spatial transcriptomic data (e.g. multiplex immunohistochemistry/immunofluorescence), however methodologies for simultaneous spatial profiling of the transcriptome and proteome are evolving.

The selection of ST technique in the lymphoma studies to date likely reflects the commercial technologies available at the time of study inception and compatibility with archived samples in FFPE or fresh frozen format. The two technologies used in these studies, Visium and GeoMx DSP, utilize NGS for sequencing and are transcriptome-wide techniques which offer an unbiased overview of the tissue milieu. However there is a trade off with lower detection efficiency of genes and a lack of single-cell resolution: 55 um per barcoded spot for Visium (analogous to 1 - 10 cells) and 700 - 800 um in GeoMx (88). While NGS-based technologies tend to allow coverage of a large tissue area, the lymphoma studies utilizing GeoMX selected a region of interest (ROI) based on morphology and protein expression. This can be advantageous in providing more efficiency in detection of transcripts within cells of interest but it also introduces bias in the data which is generated and which will not represent the tumor or TME as a whole.

## 5 Spatial transcriptomics in lymphoma: the future

Spatial transcriptomic technologies are compatible with both FFPE and fresh frozen samples which allows the retrospective analysis of tissue and, as the number of studies increases, will provide the spatially-resolved single-cell transcriptional profile of different subtypes of lymphoma and shine a light on the spatial interactions of the lymphoma cells and TME. The combination of spatial transcriptomics and deep learning models could improve pathological classification of lymphoma and identify diagnostic, prognostic or predictive markers such as individual gene markers, presence and abundance of certain cell subpopulations, and gene signatures (90).

Epigenetic alterations play a key role in the development of lymphoma through regulating gene expression and thus altering the tumor cell biological activity and the regulation of immune cell activation and infiltration within the TME (91). Epigenomics can be studied at a single cell level by chromatin accessibility profiling through techniques such as the assay for transposase-accessible chromatin using sequencing (ATAC-seq) (92), and can be combined with spatial barcoding to provide spatial epigenetic mapping (spatial-ATAC-seq) (93). Epigenetic alterations in lymphoma include those involved in DNA methylation (e.g. DNMT1), histone acetylation (e.g. CREBBP), histone methylation (e.g. EZH2) and non-coding RNA (e.g. miR-155) (94). Personalized therapies can be used to target these alterations, for example tazemetostat, an oral EZH2-inhibitor, produces durable responses in the setting of relapsed and refractory follicular lymphoma (95). The study of lymphoma using newer techniques of spatial multi-omics, including epigenomics, will therefore further improve the identification of therapeutic targets (96).

**Figure 4:**
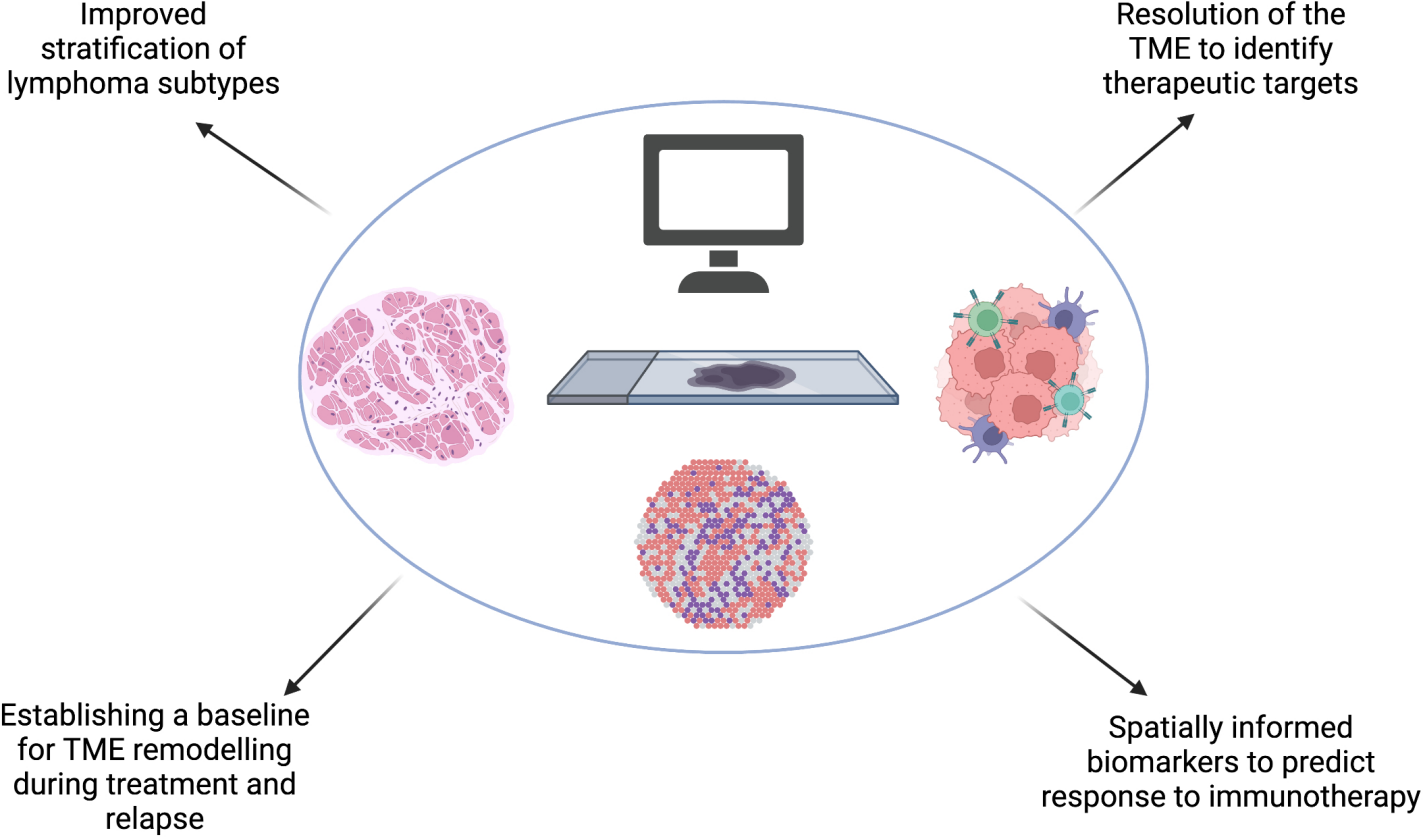
Spatial transcriptomics will improve our understanding of the spatial molecular and genetic signatures in lymphoma which could improve our existing stratification of subtypes. Spatial transcriptomic studies will identify key mechanisms which confer resistance to treatment and emergence of relapse in lymphoma, including predicting barriers to effective immunotherapy and understanding the effect of TME remodeling during treatment and at relapse to subsequent therapy. These, combined, will bring hemato-oncologists closer to the goal of precision medicine. Created with BioRender.com.

Many of the studies discussed in this article focus on the interaction between tumour cells and the TME, however studies which utilise spatial transcriptomics also provide valuable insights into lymphoma tumour cell heterogeneity. This information will identify potential for therapeutic approaches which target the spatial organization of distinct subclasses of tumour cells, for example lymphoma cell clusters which induce variable local immunosuppression at the tumor invasive margin and tumor core (97). Translational applications of spatial transcriptomic studies are summarized in **Figure 4**.

## 6 Discussion

The existing literature on spatial transcriptomics in lymphoma demonstrates that tumor survival and development rely on spatial gene expression patterns, both expressed by the lymphoma cells itself and also by the surrounding immune milieu, and findings from spatial transcriptomics are correlated to patient outcome and treatment response. Targeting of the TME in lymphoma has led to an increasing focus on immunotherapeutic strategies which have transformed the landscape of treatment options, particularly in the setting of relapsed/refractory Hodgkin lymphoma and B-cell lymphoma. However responses and duration of remissions are varied within these tumor groups and progress has been much less pronounced in T cell lymphoma, likely reflecting the varied and dynamic TME both between and within tumor groups. Many hemato-oncologists envisage a future where chemotherapy will be confined to the annals of history, with precision therapy employed across the board. To achieve this goal, we need to better understand both the profile of immune and non-immune cells within the TME and their spatial location. The latter is essential to visualize the interplay between cells within the TME and neoplastic cells, to understand the pathogenic mechanisms which lead to tumor survival, and thus to identify both potential new drug targets and new markers of clinical outcome and response to immunotherapies. Spatial transcriptomics will be an essential tool to achieve this goal.

## Author contributions

KP: Conceptualization, Investigation, Methodology, Project administration, Visualization, Writing – original draft, Writing – review & editing. ES: Investigation, Writing – original draft, Writing – review & editing. AM: Investigation, Writing – original draft, Writing – review & editing. LJ: Conceptualization, Writing – review & editing. CB: Conceptualization, Writing – review & editing.
